# *Dictyostelium*: A Model for Studying the Extracellular Vesicle Messengers Involved in Human Health and Disease

**DOI:** 10.3390/cells8030225

**Published:** 2019-03-08

**Authors:** Irène Tatischeff

**Affiliations:** Honorary CNRS (Centre de la Recherche Scientifique, Paris, France) and UPMC (Université Pierre et Marie Curie, Paris, France) Research Director, Founder of RevInterCell, a Scientific Consulting Service, 91400 Orsay, France; irene.tatischeff@upmc.fr; Tel.: +33-683-147-187

**Keywords:** extracellular vesicles, microvesicles, exosomes, oncosomes, apoptotic bodies, intercellular communication, human disease, cancer, *Dictyostelium discoideum*

## Abstract

Cell-derived extracellular vesicles (EVs) are newly uncovered messengers for intercellular communication. They are released by almost all cell types in the three kingdoms, Archeabacteria, Bacteria and Eukaryotes. They are known to mediate important biological functions and to be increasingly involved in cell physiology and in many human diseases, especially in oncology. The aim of this review is to recapitulate the current knowledge about EVs and to summarize our pioneering work about *Dictyostelium discoideum* EVs. However, many challenges remain unsolved in the EV research field, before any EV application for theranostics (diagnosis, prognosis, and therapy) of human cancers, can be efficiently implemented in the clinics. *Dictyostelium* might be an outstanding eukaryotic cell model for deciphering the utmost challenging problem of EV heterogeneity, and for unraveling the still mostly unknown mechanisms of their specific functions as mediators of intercellular communication.

## 1. Introduction

After a brief presentation of the extracellular vesicles (EVs) and of the eukaryotic microorganism *Dictyostelium*, an overview will be given about the properties of EVs and their involvement in human health and disease. Then, our pionnering work about *Dictyostelium* EVs will be presented in addition to the assets of *Dictyostelium*, as a model for studying the mammalian EVs will be discussed.

### 1.1. Presentation of the Extracellular Vesicles

Cell theory was officially formulated in 1838–1839, stating that the cell is the basic component of living organisms [1]. So the cell emerged slowly to birth, as the ultimate unit of life from the seventienth to the ninetienth century. During this time, the cell was perceived as a more complex factory, regulating its multiple biological functions by means of its many macromolecular components. As a consequence, the DNA was attributed to the major director role, orchestrating all the other components in a different set of pathways. Until recently, each cell was delimited by a membrane aimed to protect its precious content from any harmful external invasion and the extracellular medium was mostly devoted to a garbage disposal; even if a few cell-derived proteins, such as proteases or hormones already had specific intercellular functions.

One important «earthquake» in cell biology arrived insidiously, initiated by the observation that plasma contains a subcellular factor that promotes the clotting of blood [2]; two decades later, Wolf showed that this subcellular factor consists of vesicles of platelet origin, named “platelet dust” [3]. Thus, the platelet plasma membrane was no longer an impermeable border, and the cell extended its field outside the cell factory. In 1981, Trams et al. reported the exfoliation of membrane ecto-enzymes in the form of microvesicles [4]. Beside these pionneer observations, two Canadian teams worked on the maturation of sheep reticulocytes into erythrocytes over a number of years, and showed that the obsolete protein transferrin was transported outside the cells by a means of extracellular vesicles, called “exosomes”. This was also observed for the maturation of human reticulocytes, and the exosome-mediated release of obsolete cellular proteins was suggested as a general mechanism [5]. In 1999, Heijnen et al. observed that activated platelets release two types of membrane vesicles: microvesicles by surface shedding and exosomes derived from exocytosis of multivesicular bodies and alpha-granules [6]. After the first 2005 Exosome Meeting in Canada, C. Théry and G. Raposo organised the second International Exosome Workshop (IWE) [7]. Founding of an International Society, ISEV, was decided and devoted to the study of all the Extracellular Vesicles—not limited to Exosomes—with a dedicated Journal of Extracellular Vesicles, JEV, and a yearly International Congress. This was achieved during the first 2012 ISEV Meeting with about 400 participants [8,9], whereas, the 2018 ISEV meeting gathered about 1100 participants [10]. Before ISEV, another International Society on Thrombosis and Haemostasis, ISTH, was founded in 1969, more centered on Microvesicles in Health and Disease [11], but now with many aims in common. Both Societies joined for the first time at the Educational Day before the 2016 ISEV Meeting. 

### 1.2. Presentation of Dictyostelium

*Dictyostelium discoideum* was discovered in 1935 by Raper in a North Carolina (USA) forest [12], and has been widely studied ever since. For simplification, *Dictyostelium* is further used for *Dictyostelium discoideum* in this review. *Dictyostelium* is a eukaryotic amoeba at the border of the vegetal and animal kingdoms, which appeared in evolution about one billion years ago, long before mankind. In the wild, it grows on bacteria and cell divides by mitosis, but in the lab it can also grow in an axenic medium without any calf serum [13], or even in a completely defined medium [14,15] and cell divide also by mitosis. The individual growing cells are analogous to human leukocytes, with regard to their size (about 10 µm in diameter) and motility, and to macrophages with regard to their capacity for phagocytosis. In conditions of complete starvation, these *Dictyostelium*, “animal-like” cells, first experience a primitive multicellular aggregation, followed by a simple differentiation into two main “vegetal-like” cells, stalk cells, and spores. Aggregation tests in (3.5 cm in diameter) tissue culture Petri dishes with 2 × 10^6^ adhering cells in 1 mL KK_2_ buffer depict cAMP-driven chemotaxis, with cell elongation within about 6 h of starvation, and further formation of nice aggregation figures, either in stars or in spirals. When being at an air-interface in (4.1 cm in diameter) differentiation tests, each complete aggregate gives rise in about 24 h from initation of starvation, to a visible so-called fruiting body, inholding (1/3) stalk cells, programmed to death and organised into a stalk, bearing a small balloon, including the (2/3) spores, programmed to further life by germination into new amoebae, when recovering normal nutrition conditions [16]. Thus, for this primitive eukaryotic species, growth and differenciation are well separated biological processes, and complete starvation induces the transformation of individual cells (about 10 µm in diameter) into a visible fruiting body (a few tenth of mm high). A. Einstein, watching J. T. Bonner’s 1940 video about this slime mold development, in Princeton (USA), was impressed by this amazing microorganism [17].

Besides its quite noticeable lifestyle, *Dictyostelium* possess many other assets. Its small (3.4 × 10^7^ bp) genomic DNA has been completely sequenced [18], and covers six chromosomes, with a 90% efficient transcription into about 12,500 genes. By comparison, the human (about 10^9^ bp) genomic DNA is 10% transcribed, with only about twice as many genes as *Dictyostelium*, which is devoted with some genes analogous to some important human genes. *Dictyostelium* cells also harbor mitochondria with a fully sequenced genome [19], and plasmids. More details about *Dictyostelium* can be found in the well documented website (https//www.dictybase.org), and an increasing number of specific strains and plasmids can be ordered from the *Dictyostelium* Stock center.

*Dictyostelium* has been chosen in 1999 by the NIH (USA), as a new non-mammalian model organism for biomedical research. In 2011, R. Escalante gathered the works from many labs to present *Dictyostelium* as a model for human disease [20]. As stated by S. Bozzaro [21]: “This model organism has been particularly useful for the study of cell motility, chemotaxis, phagocytosis, endocytic vesicle traffic, cell adhesion, pattern formation, caspase-independent cell death, and, more recently, autophagy and social evolution. It has proven to be a powerful genetic and cellular model for investigating host–pathogen interactions and microbial infections, for mitochondrial diseases, and for pharmacogenetic studies. The *D*. *discoideum* genome harbors several homologs of human genes responsible for a variety of diseases, including Chediak-Higashi syndrome, lissencephaly, mucolipidosis, Huntington disease, IBMPFD—that can affect the muscles, bones, and brain—and Shwachman-Diamond syndrome. The study of some of these genes has provided new insights on the mechanism of action of the encoded proteins and, in some cases, on the defect underlying the disease”.

## 2. Overview of the Extracellular Vesicles

Here are recapitulated the main EVs characteristics and reported biological functions, with no details about the few already elucidated mechanisms, which have to be searched in more specialized reviews.

### 2.1. Definition and Characteristics of the Extracellular Vesicles

These days, the EV field is extraordinarily complex, due to the huge diversity of their observations. After the pioneering work of Wolf on platelets [3], Apoptotic bodies, with a size up to 5 µm, released by cells dying by apoptosis [22] were the first EVs to be observed. Microvesicles or Ectosomes, previously named Microparticles, originated mainly from human body fluids, such as blood, plasma and urine, and were generally observed in a clinical environment. With a size between 100 nm and 1 µm, they were rather easy to prepare by low differential centrifugation, and to be characterised by their membrane antigens, mostly by using specific antibodies and normal fluorescence flow cytometers, at least above their 300 nm resolution threshold. These two EV classes shared a phosphatidylserine (PS) transfer from the inner to the outer lipidic bilayer, and a common biogenesis, corresponding to the shedding of pieces of the cell plasma membrane (PM), and embedding different macromolecular cargoes. Exosomes, and Exosome-like EVs, such as Prostasomes, were smaller, with a size between 40 and 150 nm, and were first mostly prepared by differential centrifugation, ending with two final steps of ultracentrifugation at 100,000g [23]; they were mostly characterised by western blots and proteomics in a biological environment. Their biogenesis were linked to endocytic processes through the cells until their accumulation into multivesicular bodies (MVBs), partly fusionning with the PM for the release of their inner vesicles outside the cells as EVs. More recently, the EV family increased with the appearance of Oncosomes, shed from the PM of some—not only tumor—cells, with a size up to 10 µm [24,25,26,27], therefore the EV family is always increasing.

All EV main classes differ first by their size, 40–150 nm for the exosomes, 100 nm–1 µm for the microvesicles, up to 5 µm for the apoptotic bodies and up to 10 µm for the oncosomes. However, these different EVs cannot be confidently discriminated by size, due to their partial overlapping. EVs differ also by their biogenesis: microvesicles and oncosomes originate from the shedding of pieces of plasma membrane; apoptotic bodies originate from a lesser known ultimate cell reconditioning, whereas exosomes experience an intracellular traffic through the well-known endocytic pathways. Besides differences in size and biogenesis, the most important characteristics of the different EVs are their respective cargoes, with defined contents of molecular components (proteins, lipids, nucleic acids and metabolites), giving them different densities. The known EV macromolecular compounds have been classified in three databases [28,29,30,31]. However, EVs can neither be discriminated by their quite different cargoes, due to the absence of some true class-specific biomarkers. Almost all cells, whatever their kingdom, Archeabacteria, Bacteria or Eukaryotes, are physiologically releasing EVs [32,33], suggesting that this might also be the case for the Last Universal Common Ancestor (LUCA) [34], which is the most recent organism from which all modern cells derive, and that EV release might indeed be of the utmost importance for cell biology. This is also the case for the different cells of the human body and the various body fluids, although with varying amounts. Therefore, the EV landscape seems to be a “continuum” of different EVs, more or less well classified into four main EV populations, as were the spectral lines of the H atom before Niels Bohr’s atomic theory. Many reviews have been devoted to the classification of EVs [35,36,37]. In the absence of a general consensus about EV nomenclature [38], the International Society ISEV advocates the general use of EVs, whatever the EV class used in the current scientific papers.

On the other hand, EVs are endowed with important biological functions, which will be summarized below. Moreover, EV concentrations are generally increasing in many diseases with specific changes of their cargoes, especially in human cancers. Therefore, tumor cell-derived EVs might be promising as biomarkers and even as therapeutic agents for drug delivery. These points of interest will also be detailed below. After a rather slow and messy emergence until 2012, the EV field is now experiencing a tremendous increase of interest in both biology and medicine, as shown by the fast growing EV publication rate [39].

### 2.2. Extracellular Vesicles and Intercellular Communication

Besides their characterization by size, biogenesis and cargo contents, the EV biological properties, although still mostly unknown, have been progressively discovered [40,41]. One of the earliest observations was the externalisation of the transferrin receptor from sheep reticulocytes in vitro [42]. It was later suggested as a general exsosomal process for shedding membrane proteins [5]. Already in 1996, it was mentioned that B-lymphocytes secrete antigen-presenting vesicles [43]. The roles of membrane vesicles and exosomes in immune responses were further elucidated [44,45,46]. Microvesicles also participate in important biological processes, such as the surface–membrane traffic and the horizontal transfer of proteins and RNAs among neighbouring cells. In 2006, whereas the horizontal transfer of DNA by the uptake of apoptotic bodies was already a known process [47], J. Ratajczak et al. stressed that membrane-derived vesicles were important mediators of cell-to-cell communication [48], and they brought evidence for the horizontal transfer of mRNA and protein delivery by embryonic stem cell-derived microvesicles [49]. Valadi et al. reported a novel mechanism of genetic exchange between cells, mediated by the exosome transfer of mRNA and miRNA [50]. Among other beneficial influences of exosomes, one can mention their communication of protective messages during oxidative stress [51]. Nowadays, the EV active participation in intercellular communication is convincingly claimed [35,48,52,53,54,55]. As stated by Camussi et al. [56] “even though the exact physiological role of EVs remains to be elucidated, it is becoming clear that they may transfer proteins, receptors, bioactive lipids, messenger ribonucleic acid (mRNA), and micro-RNA (miRNA) from the cell of origin to the recipient cell, which may modify their phenotype and functions”. The physiological roles of exosomes is probably important for monitoring body homeostasis during health, but is less well-studied than their pathological roles in many human diseases. The exosome-like vesicles prostasomes, originating from the prostate, represent an exception, as their influence in normal human reproduction was one of the earliest works of interest about EVs [57]. During normal pregnancy, placental vesicles have been shown to have a wide range of functional activities, transferring a variety of bioactive molecules into the maternal circulation [58]. Recently, EVs have also been implied in senescence and aging [59]. However, the EV-mediated intercellular communication is a “double-edge sword”, as cells can release prions in association with exosomes [60], and exosomes can also mediate the functional delivery of viral miRNA [61], whereas microvesicles too can contribute to viral infection [62].

### 2.3. Extracellular Vesicles and Human Diseases

**Figure 1:**
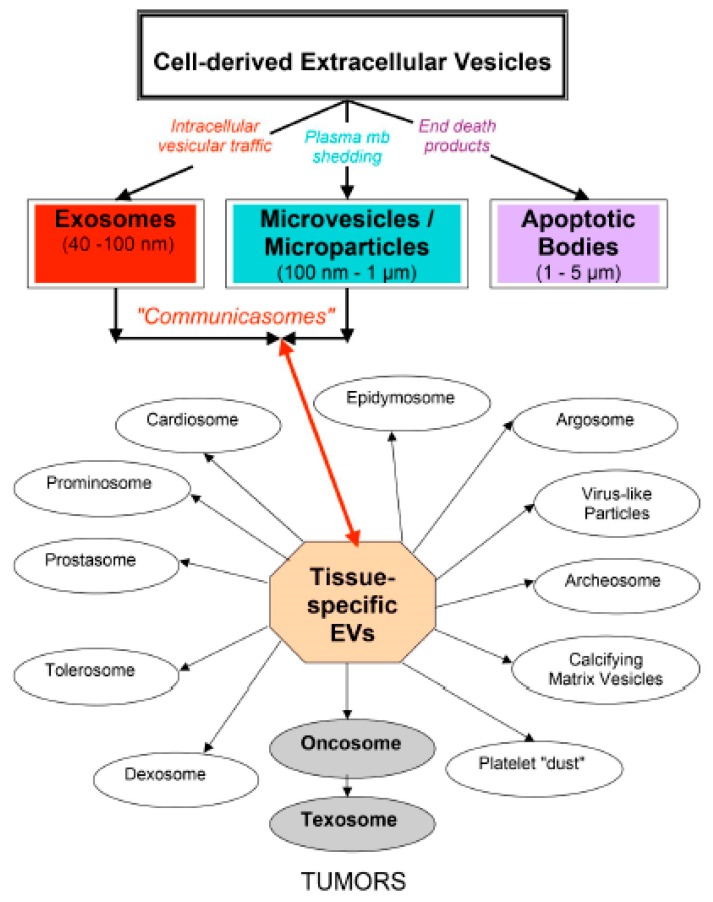
Complexity of eukaryotic EVs. Following their respective biogenesis, three main classes of cell-derived EVs are now recognized: exosomes, microvesicles/microparticles and apoptotic bodies. A tissue-specific EV classification is also shown to point out their interest for the medical field.

Figure 1 (taken from [63]) shows the complexity of the cell-derived EVs. This tissue-specific EV classification points out their interest in the medical field. As stressed more recently [27], only large oncosomes are released by some tumor cells, whereas oncosomes might also be releasd by non-tumor cells. The possibility of using EVs as biomarkers and even therapeutics in many human diseases has sustained the increasing interest for EV research [39]. This approach was first experimented with the microvesicles, as detailed in [11]. Shedding vesicles play a role in inflammation and thrombosis [64], in vascular diseases [65] and in pre-eclampsia versus normal pregnancy [66]. Pregnancy affords a unique opportunity for a comparative EV study in normal physiology and disease [58]. In addition, microvesicles have important physiological roles in coagulation in vivo, by mediating the coordinate contribution of platelets, macrophages, and neutrophils [67]. Endothelial-derived microparticles are said to be biological conveyors at the crossroad of inflammation, thrombosis, and angiogenesis [68]. EVs have deleterious effects (pro-inflammatory, pro-angiogenic, pro-thrombic, vascular dysfonction, and pro-apoptotic), as well as beneficial effects (anti-inflammatory, post-ischemic angiogenesis, and anti-apoptotic), in cardiovascular pathologies, depending on the molecules they carry [69], (p. 397). Epigenetic changes induced by EVs have been particularly studied in the context of immunology, cancer and stem cell biology [56].

Table 1 shows the suggested topics for abstract classifications of ISEV 2018 [10], stressing the current huge human medical involvement of EVs. Presently, EVs are involved in the immune system, in cardiovascular diseases and vascular disorders, in reproduction and pregnancy, and in the nervous system (blood-brain-barrier)**.** They are also involved in tissue injury, repair and remodeling; in viral, bacterial, fungal, and parasitic infections; in acute and chronic inflammatory disorders; in stem cells and in cancer, especially in tumor immunology, angiogenesis, and metastasis; as well as in neurodegenerative diseases [70].

### 2.4. Extracellular Vesicles and Cancer

Cancer is by far the most studied human disease under the EV light. However, no unique cancer-specific pathway has yet emerged. Each of the most common human cancers behaves like a specific illness and develops its own panel of various tumor cells-derived EVs, with specific compositions and timely influences on the tumoral near or distant environment. Many papers are devoted to EVs with a given specific human cancer, but are out of the scope of this review. Each main EV class can be an actor implied in cancerogenesis, but its relative importance, compared with the ones of the other EVs classes can vary for different tumors. Microvesicles have important pathological roles as mediators of intercellular communication in cancer [71] and in tumor progression by novel microenvironment modulators [72], facilitating the spreading and release of cancer cells to generate metastases [67].

The general properties inventoried for tumor EVs are related either to the transport and intercellular transfer of active compounds, such as oncogenes, functional miRNAs, tumor suppressor proteins, and antitumoral drug resistance proteins, or to biological functions, dealing with modified immunological properties and angiogenesis, or specific organ-targeted metastasis. Many papers or recent reviews summarize these observed properties of tumor EVs [25,26,63,73,74,75,76,77,78,79,80,81].

### 2.5. Extracellular Vesicles, Drug Delivery, and Cancer Therapy

The more achieved EV therapy corresponds to the early use of dendritic cell-derived exosomes, as a novel cell-free vaccine for the eradication of murine tumors [82], which is now efficient as an immunotherapy treatment for some human tumors.

In 2013, Ohno et al. overviewed the potential roles of exosomes and microvesicles with respect to clinical diagnosis and disease pathogenesis [83]. Beside their great potential as diagnostic and pronostic biomarkers, EVs are promising drug delivery systems, following “the Trojan exosome hypothesis”, according to which exosomes might be good candidates for crossing the rather impervious cell biological barriers for drug delivery [84]. This has been applied to the drug delivery of RNAi [85] and siRNA [86]. Although also being Trojan horses for viral infections [87], EVs are now considered unique intercellular delivery vehicles [88]. Potential applications of EVs were suggested in cancer diagnosis, prognosis, and epidemiology [89]. Basic and clinical scientists joined to summarize recent developments and the current knowledge of EV-based therapies. Strategies to promote the therapeutic application of EVs in future clinical studies were addressed [90]. This has been recently actualized for the development of best practice models for EV therapies [39,91], which might bring future improvements in cancer care [92].

### 2.6. Challenges Faced for Therapeutic Use of Extracellular Vesicles

EVs are quite appealing for future theranostics (diagnosis, prognosis, and therapy) of human diseases, and especially cancer. However, no efficient clinical use is possible yet, due mainly to some challenging unsolved problems, i.e., the absence of the consensus in regard to the EV nomenclature [38], the absence of standardisation of the EV measurements at a large scale [93], and the crucial unsolved problem of EV heterogeneity [94,95,96,97]. Besides the intrisic EV heterogeneity, heterogeneity is ever present in the human body. Many different types of healthy human cells have the potential to secrete EVs into bodily fluids, with the possible increase and modification, and contribution from sick cells; futhermore, an important microbiome (10 times more bacteria than human cells) brings its own capacity to externalize EVs, due to the universal process of EV secretion [33]. The general estimation of about 100 times more viruses than human cells, their size analogy with exosomes and the recent observation of SVF-derived gesicles [98] suggest that viruses too, might contribute to an increase in the so-called “mammalian EVs”.

The EV field is complex not only by definition, as detailed above, but also greatly depends on the methods used for their preparation—of which the numbers are increasing with time. Beside the long used unique centrifugation protocols, there are some new filtration processes and some available commercial kits, based on EVs precipitation [97,99]. When working with body fluids, pre-analytical conditions before EVs preparation are also quite important. The standardisation of EV measurements is an important challenge to solve a wide inter-organisation comparison of the different EV measurements, especially in clinical set-ups. Recently, the technical challenges for working with EVs were discussed, and some possible options to overcome them were suggested [93,99]. In 2014, ISEV provided researchers with a minimal set of experimental requirements for the definition of EVs and their functions [100], which was further updated [101,102]. The deciphering of EVs into specific subpopulations, linked to their specific biological functions, remains one of the biggest challenges to solve, before using the great potentialities of EVs in the theranostics of many human diseases, including cancer.

It is to be noticed that up to now, many EV studies have been performed directly at the clinical level in human body fluids, or in vitro on different human disease-related cell lines. A simple eukaryotic EV model is urgently needed, to help solve some of the remaining challenges, which are too complex to be worked out directly at the human level. *Dictyostelium* might be such an appealing eukaryotic EV model, as argumented in the next part of this paper.

## 3. *Dictyostelium* as a Model for Studying Mammalian Extracellular Vesicles

### 3.1. Discovery of Dictyostelium Extracellular Vesicles by Serendipity

*Dictyostelium* EVs were unknown until our observation in 1998 [103]. This was the result of many years of unprogrammed research, favored by serendipity, as outlined below.

After a PhD in physics, I met *Dictyostelium* during a 1977 sabbatical at the University of British Columbia (UBC, Vancouver, Canada). Following a method, said to discriminate the rigidity of the cell plasma membrane of leukemic cells from the one of the normal cells [104,105], I used a newly achieved UBC home-made fluorescence polarization set-up with an analogic photomultiplier detection with *Dictyostelium* cells [106]. When coming back to the Curie’s lab in Paris, I decided to switch my research towards biology with *Dictyostelium*. I was helped greatly by P. Brachet (Pasteur Institute, Paris) for introducing this wonderful microorganism into a physics environment. I was very lucky to manage working with it, as a CNRS scientist until 2003, and, then until 2013, as an Honorary UPMC Research Director.

Our first goal was to build an automated set-up for fluorescence polarization measurements, but with the photon counting technology, previously elaborated in our team. We managed to measure a higher plasma membrane rigidity for *Dictyostelium* cells during early development, compared to one of the growing cells [107]. But we also observed a quite unexpected release of fluorescent compounds into the KK_2_ phosphate starvation buffer, accompanying early aggregation of the cells in suspension [108]. This was the beginning of a fruitful investigation headed by R. Klein, who deciphered the GTP catabolism, giving rise to the pteridine pathway, and ending with the formation of a specific pterin, named *Dictyopterin* [109].

We improved our set-up in order to measure the known spontaneous oscillations of aggregation-competent cells in suspension [110]. In parallel, I remained fascinated by watching via light microscopy, and taking pictures of *Dictyostelium* growing cells, and of cells further starvation-induced into aggregation and differentiation, after incubation of the cells with many different compounds, or without comparison. The growth of axenic *Dictyostelium* cells was also measured in a complete defined medium [14], or in the same medium without 5 × 10^−7^ M folic acid, and their respective developments were compared.

When becoming more familiar with *Dictyostelium,* I asked the first “funny” question: Why do *Dictyostelium* cells never get cancer? *Dictyostelium, strain* (Ax-2) growing cells were incubated with either the main carcinogenic compound of tobacco smoke, benzo (a) pyrene, B(a)P, or with its non-carcinogenic isomer benzo (e) pyrene, B(e)P. The current theory was that only B(a)P was metabolized into a diol-epoxide compound, which initiated the tumoral process. But with *Dictyostelium* cells, we noticed that the shape recognition of the two BP isomers occurred before any metabolization: only the fluorescent harmful B(a) P was released into the KK_2_ starvation medium. This was presented at the thirteenth International Symposium on Polynuclear Aromatic Hydrocarbons [111], but was futher rejected for publication (“nothing to do with cancer” for cancer journals/”too much dealing with cancer” for biological journals). As M. Gottesman was then deciphering the P-glycoprotein (P-gp)-mediated multidrug resistance [112,113,114], I asked whether such an ABC transporter might exist in *Dictyostelium* cells and explain their already recognized high resistance against many structurally different xenobiotics. By the help of a medical collaboration with A.-M. Faussat, using a mouse antibody against P-gp, we showed that, in fact, *Dictyostelium* cells harbor a P-gp with the same 170 kDa mass as the human P-gp [115]. However, by means of another collaboration with G. Lizard in Dijon, we showed, by using flow cytometry, that this P-gp was non-functional [116]. At the same time, the multidrug landscape became more complex with the appearance, beside the P-gp, of the multidrug resistance protein (MRP), and of the lung resistance protein (LRP), acting at the nuclear membrane level, so we have temporarily left this research field.

In order to get a homogeneous cell population in a given state of the cell cycle, before initiating starvation-induced development, we reproduced a simple method for synchronizing growing *Dictyostelium* cells [117]. We needed to control the cell DNA amount in a timely manner, therefore we chose the widely used DNA-specific Hoechst 33,342 (HO342) vital stain for labeling aliquots of the synchronized growing cells, as a function of time. Unexpectedly, *Dictyostelium* cells were completely resistant to HO342 vital staining, and when watching the cells once more with a light fluorescence microscope, we noticed that the cells were surrounded with numerous fluorescent particles. These particles turned out to be detoxyfing vesicles, inholding HO342, which was thus prevented to reach its nuclear DNA target. Here, was the long-searched resistance mechanism of *Dictyostelium* cells. Morover, the control experiment without HO342 labeling showed that extracellular vesicles were present, not only as a detoxyfing mechanism, but as a common physiological process [103]. This was the beginning of the fruitful EV story of *Dictyostelium* cells.

### 3.2. Story of the Dictyostelium Extracellular Vesicles (1998–2013)

*Dictyostelium* cells vitally stained with the DNA-specific dye, HO342, released fluorescent material in their culture medium. By means of lipid analysis and electron microscopy, we demonstrated the vesicular nature of this material, which turned out to be organelles of about 100 to 300 nm, surrounded by a lipid bilayer envelope. Furthermore, we observed that proteins and nucleic acids, both DNA and RNA, associated with these extracellular vesicles, independently of HO342 vital staining. The main vesicular DNA component exhibited a size > 21 kb, and its association with vesicles in physiological growth conditions, and not concomitant with any programmed cell death, suggested a possible involvement of these EVs in a more general intercellular mechanism, than the newly observed cellular resistance to vital HO342 DNA-staining [103].

The second important observation was that the HO342-transporting *Dictyostelium* EVs were able to completely overcome the natural resistance of *Dictyostelium* cells to HO342 vital staining and to transport the dye into their DNA nuclear target. This was true not only for *Dictyostelium cells*, but also for human leukemic resistant cells, K562r, as shown in a fluorescence study [118]. This gave rise to an European patent, extended to the USA and Canada, advocating for the use of *Dictyostelium* EVs for tranferring a molecule of interest to an eukaryotic cell [119]. This EV property was also tested with hypericin (Hyp), which is used for photodynamic therapy in some cancers, and is a very hydrophobic molecule, quite different from HO342. Here again, *Dictyostelium* detoxifying EVs, inholding Hyp, were able to transfer the drug to its known target, i.e., the Golgi, into living Hela cells [120]. Co-internalization of magnetic nanoparticles and fluorescent dextran in *Dictyostelium* cells demonstrated the possibility to design multifunctional biovesicles, carrying both magnetic agents and therapeutic molecules, for targeting a tumor area by means of magnetic attraction [121]. The *Dictyostelium* cells derived EV strategy for drug delivery has been further detailed [122]. In parallel, the new EV-mediated detoxifying mechanism, discovered with *Dictyostelium*, was presented at the International Exosome Workshop (IWE) [7], and further suggested to be involved as a new multidrug resistance mechanism, at work during the failure of antitumoral treatment by chemotherapy [123].

Our whole study about *Dictyostelium* EVs was summarized in a poster for the first 2012 ISEV Meeting [9]. The assets of the non-pathogenic micro-organism *Dictyostelium discoideum* have been stressed, in order to promote it as an interesting model for the study of eucaryotic EVs [124]. *Dictyostelium* EVs were also used to elaborate a new EV characterization method, Raman Tweezers Microspectroscopy (RTM), with a home-made set-up, in order to measure the global molecular compostion of a single (or a few) EV(s), without any labeling. The global molecular compositions of *Dictyostelim* EVs, either derived from growing cells or from aggregating cells were found to be quite different [125]. A thorough study of this technology has recently been published [126], which will potentially be useful for the molecular characterization of single (or a few) EV(s) in pre-defined EV subpopulations.

### 3.3. Dictyostelium Could be an Outstanding Eukaryotic Model for Studying Mammalian Extracellular Vesicles

All the intracellular and extracellular vesicles present a characteristic lipid composition and organization that governs their formation, targeting, and function. The liquid crystalline structure of lipids plays an essential role in their function as biological nanovehicles of information [127].

One asset of *Dictyostelium* lies in its easy manipulation in conditioned media experiments. *Dictyostelium* cells are able to grow in agitated suspensions or as adhering cells. In simple tissue culture flasks, one can easily control the whole *Dictyostelium* life cycle, i.e., the growth, as well as starvation-induced aggregation and differentiation into fruiting bodies. The corresponding conditioned media of this unique eukaryotic both in vitro and in vivo cell model could be easily collected. With the current progress for EV preparation and characterization, it would be possible to address the important challenge of EV heterogeneity (exosomes, microvesicles, apoptotic bodies, oncosomes, and their respective subpopulations) in this simple cell model. The further easy design of conditioned media experiments might offer the possibility to study the respective biological functions of these different pre-defined EV subpopulations.

Another suggestion would be to reconsider our experiment about the inhibition of multicellular development, which switches the cell death of *Dictyostelium* towards mammalian-like unicellular apoptosis [128], by questioning the influence of EVs. Briefly, a conditioned medium was obtained by first starving a 4 × 10^7^ c/mL *Dictyostelium* cell population in agitated suspension during 22h in a KK_2_ phosphate buffer (pH 6.8). After getting rid of the cells by centrifugation, the conditioned medium was obtained and named t22, linked to the time of cell starvation in suspension. When performing the usual aggregation test with 2 × 10^6^ c/mL, new growing *Dictyostelium* cells in 1 mL of this t22 conditioned medium, their aggregation was completely blocked and all the cells were induced to an apoptotic death, with many characteristics of human apoptosis, including the mitochondrial release of an apoptosis-inducing factor [129]. A t8 conditioned medium, obtained after only 8 hours of starvation did impair the aggregation of new cells, but without further inducing cell apoptosis. EVs were observed in the t22 conditioned medium, but not studied at that time. One can now wonder whether EVs were involved in these observed biological effects, what was the EV differences between the two (t8/t22) conditioned media, and whether a late-appearing EV subpopulation might be able to induce *Dictyostelium* cells into a human-like apoptotic death.

The significance of *Dictyostelium* for deciphering EV biological functions was nicely demonstrated recently [130]. Despite its one billion year ancestral position in evolution, *Dictyostelium* was brought to scientific discovery only in 1935 [12]. Its splendid patterns of starvation-induced aggregation remained for more than three decades under the mysterious Acrasiale power, then the orchestrating role of c-AMP was discovered in J. T. Bonner’s lab in 1969 [131]. Many labs were further involved in deciphering the c-AMP mechanism, but nearly five decades passed before EVs entered into this chemotaxis process [130,132]. C. A. Parent et al. have nicely shown that a polarized *Dictyostelium* cell migrating towards an agregation center expells EVs from its rear part, and that these EVs contain all the machinery (Adenylate Cyclase and ATP) to self-assume the c-AMP biosynthesis. Moreover, among the 68 *Dictyostelium* cell pumps, 13 remain associated with the EVs and one is specially devoted to quantitatively expell the newly formed c -AMP for attracting the other following *Dictyostelium* cells. The elucidation of the chemotaxis mechanism in *Dictyostelium* cells might help to clarify the exosome-promoted chemotaxis of cancer cells [133]. To my knowledge, it is only the second observation, beside the one concerning the EV-maturation of miRNAs [134], showing that EVs are not only conveyers of important macromolecular components, involved in their now widely recognized functions in intercellular communication, but that they also can act as cell-independent autonomous biological entities. This shows an appealing future for *Dictyostelium* EVs, and the long accumulated knowledge about this eucaryotic cell model (www.dictybase.org) might help to further elucidate this quite new EV biological function.

## 4. Conclusions

For the past decade, cell biology has entered “a new galaxy” by extending its research field beyond the cell plasma membrane and discovering the huge power of extracellular vesicles in intercellular communication. However, this extracellular vesicle biology is only in its infancy, and many challenging questions remain to be solved, before efficiently using EVs for the theranostics of human diseases, including cancer.

Contrary to the about 10 years working time for developing an efficient pharmaceutical compound, which are dealing with in vitro and in vivo tests before reaching the clinics, the current EV in vivo experiments are still scarce. All the cell lines linked to a given cancer type f. ex. are interesting tools for searching specific biomarkers, but are relatively too simple to catch an important cancer-specific mechanism. On the opposite, EV clinical research on human bio fluids, being highly complex, is like “looking for a needle in a haystack”. The lack of simple eukaryotic models for deciphering the EV-mediated biological functions greatly hampers the current knowledge about EV-mediated functions in human health and disease. Dictyostelium discoideum, which was recognised in 1999 by the National Institutes of Health (NIH, USA), as a new interesting model for biomedical research, might help to bridge the current gap of the human EV knowledge between in vitro and clinical research.

## Figures and Tables

**Table 1:** Topics for Abstracts Classification of ISEV 2018.

EV biogenesis (from prokaryotes to eukaryotes) EV in environment and cross kingdom communication Cellular and organ targeting of EVs EVs in cellular differentiation & organ development EVs and the immune system EVs in the nervous system (blood-brain-barrier) EVs in reproduction & pregnancy EVs in tissue injury & coagulation EVs in tissue repair & remodeling EVs in tumor immunology EVs in tumor angiogenesis EVs and stem cells (including cancer) EVs in tumor metastasis EVs in cancer (except metastasis, immunology, angiogenesis, stem cells) EVs in acute and chronic inflammatory disorders EVs in diseases of the nervous system EVs in cardiovascular diseases and vascular disorders EVs, viruses, and viral infections EVs in parasitic, bacterial and fungal infections EV-based cancer Biomarkers EV-based non-cancer Biomarkers EV-inspired therapeutics and vaccines Analysis of EVs in body fluids; preparative studies, spike-ins etc EV proteomics & lipidomics EV transcriptomics Novel developments in EV isolation Novel developments in EV characterization
